# Essential Oil Characterization of *Thymus vulgaris* from Various Geographical Locations

**DOI:** 10.3390/foods5040070

**Published:** 2016-10-27

**Authors:** Prabodh Satyal, Brittney L. Murray, Robert L. McFeeters, William N. Setzer

**Affiliations:** 1 Alchemy Aromatic LLC, 621 Park East Blvd., New Albany, IN 47150, USA; prabodhsatyal@gmail.com; 2 Department of Chemistry, University of Alabama in Huntsville, Huntsville, AL 35899, USA; blm0029@uah.edu (B.L.M.); robert.mcfeeters@uah.edu (R.L.M.)

**Keywords:** chiral gas chromatography, mass spectrometry, hierarchical cluster analysis, antifungal activity, enantiomeric distribution

## Abstract

Thyme (*Thymus vulgaris* L.) is a commonly used flavoring agent and medicinal herb. Several chemotypes of thyme, based on essential oil compositions, have been established, including (1) linalool; (2) borneol; (3) geraniol; (4) sabinene hydrate; (5) thymol; (6) carvacrol, as well as a number of multiple-component chemotypes. In this work, two different *T. vulgaris* essential oils were obtained from France and two were obtained from Serbia. The chemical compositions were determined using gas chromatography–mass spectrometry. In addition, chiral gas chromatography was used to determine the enantiomeric compositions of several monoterpenoid components. The *T. vulgaris* oil from Nyons, France was of the linalool chemotype (linalool, 76.2%; linalyl acetate, 14.3%); the oil sample from Jablanicki, Serbia was of the geraniol chemotype (geraniol, 59.8%; geranyl acetate, 16.7%); the sample from Pomoravje District, Serbia was of the sabinene hydrate chemotype (*cis*-sabinene hydrate, 30.8%; *trans*-sabinene hydrate, 5.0%); and the essential oil from Richerenches, France was of the thymol chemotype (thymol, 47.1%; *p*-cymene, 20.1%). A cluster analysis based on the compositions of these essential oils as well as 81 additional *T. vulgaris* essential oils reported in the literature revealed 20 different chemotypes. This work represents the first chiral analysis of *T. vulgaris* monoterpenoids and a comprehensive description of the different chemotypes of *T. vulgaris*.

## 1. Introduction

*Thymus vulgaris* L. (Lamiaceae) is an evergreen herb native to the southern Europe and the Mediterranean [1]. The plant has been used since ancient times as a culinary ingredient, to add flavor to cheeses [2,3] and liqueurs [4,5], and to flavor meats such as rabbit, boar, and lamb [6]. Today it is a common component of *bouquet garni* [7] and of *herbes de Provence* [8]. In addition to its use in foods, *T. vulgaris* is a well-known herbal medicine that has been used for thousands of years to treat alopecia, dental plaque, dermatophyte infections, bronchitis, cough, inflammatory skin disorders, and gastrointestinal distress [9]. The major constituents of commercial *T. vulgaris* essential oil are thymol (23%–60%), γ-terpinene (18%–50%), *p*-cymene (8%–44%), carvacrol (2%–8%), and linalool (3%–4%) [10]. *T. vulgaris* oil as well as thymol have shown antibacterial, antifungal, and anti-inflammatory effects, accounting for the medicinal uses of *T. vulgaris* [9]. There are, however, numerous varieties and cultivars of *T. vulgaris*; Tropicos lists nine subspecies and varieties of *T. vulgaris* [11]. As many as 13 different chemotypes of *T. vulgaris*, based on the predominance of particular monoterpenoids in the essential oils, have been identified [12,13,14,15]. In this work, we present the essential oil compositions, including monoterpenoid enantiomeric compositions, of four different chemotypes of *T. vulgaris* essential oils from Europe. In addition, a hierarchical cluster analysis has been carried out to elucidate/delineate the various chemotypes of *T. vulgaris*, and we have examined the antifungal properties of three essential oils from Europe.

## 2. Materials and Methods

### 2.1. Plant Material

*Thymus vulgaris* #1, (linalool chemotype), *T. vulgaris* #3, (geraniol chemotype), *T. vulgaris* #4, (sabinene hydrate chemotype), and *T. vulgaris* #5, (thymol chemotype) were collected in May 2015, from Nyons, France (44°21′37″ N, 5°8′23″ E), Jablanicki, Serbia (42°59′43″ N, 21°55′4″ E), Pomoravje District, Serbia (43°58′0″ N, 21°15′0″ E) and Richerenches, France (44°21′37″ N, 4°54′47″ E), respectively. The plants were identified by Julien Abisset (Natural Essential SA, France). The air-dried aerial parts of each *T. vulgaris* sample were subjected to steam distillation for 3 h. After decanting and drying of the oils over anhydrous sodium sulfate, they were stored under refrigeration (−4 °C) until analysis. Aerial parts of *T. vulgaris* #1 produced 1.6% yield of essential oil (EO), *T. vulgaris* #3 produced 1.2% yield, *T. vulgaris* #4 produced 1.0% yield, and *T. vulgaris* #5 produced 1.5% yield. 

### 2.2. Gas Chromatography—Mass Spectrometry (GC-MS)

The essential oils of *T. vulgaris* chemotypes were analyzed by GC-MS using a Shimadzu GCMS-QP2010 Ultra operated in the electron impact (EI) mode (electron energy = 70 eV), scan range = 40–400 amu, scan rate = 3.0 scans/sec, and GC-MS solution software. The GC column was a ZB-5 fused silica capillary column with a (5% phenyl)-polymethylsiloxane stationary phase and a film thickness of 0.25 μm. The carrier gas was helium with a column head pressure of 80 psi and flow rate of 1.37 mL/min. Injector temperature was 250 °C and the ion source temperature was 200 °C. The GC oven temperature program was programmed for 50 °C initial temperature, temperature increased at a rate of 2 °C/min to 260 °C. A 5% *w*/*v* solution of the sample in CH_2_Cl_2_ was prepared and 0.1 µL was injected with a splitting mode (30:1). Identification of the oil components was based on their retention indices determined by reference to a homologous series of *n*-alkanes, and by comparison of their mass spectral fragmentation patterns with those reported in the literature [16], and stored in our in-house MS library.

### 2.3. Chiral Gas Chromatography—Mass Spectrometry

Chiral analysis of the essential oils was performed on a Shimadzu GCMS-QP2010S operated in the EI mode (electron energy = 70 eV), scan range = 40–400 amu, scan rate = 3.0 scans/s. GC was equipped with a Restek B-Dex 325 capillary column (30 m × 0.25 mm ID × 0.25 μm film). Oven temperature was started at 50 °C, and then gradually raised to 120 °C at 1.5 °C/min. The oven was then raised to 200 °C at 2 °C/min and held for 5 min. Helium was the carrier gas and the flow rate was maintained at 1.8 mL/min. Samples were diluted 3% *w*/*v* with CH_2_Cl_2_ and then a 0.1 µL sample was injected in a split mode with a split ratio of 1:45. 

### 2.4. Hierarchical Cluster Analysis

A total of 81 *T. vulgaris* essential oil compositions from the published literature, as well as the four compositions from this study were treated as operational taxonomic units (OTUs). The percentage composition of 33 major essential oil components (thymol, *p*-cymene, γ-terpinene, linalool, carvacrol, geraniol, β-caryophyllene, *cis*-sabinene hydrate, borneol, α-pinene, terpinene-4-ol, myrcene, 1,8-cineole, α-terpineol, camphene, α-terpinyl acetate, α-terpinene, camphor, limonene, β-pinene, geranyl acetate, α-thujene, geranyl formate, β-cyclocitral, *cis*-verbenol, *trans*-sabinene hydrate, 1-octen-3-ol, thymol methyl ether, caryophyllene oxide, carvacrol methyl ether, bornyl acetate, thymoquinone, and α-humulene) was used to determine the chemical relationship between the various *T. vulgaris* essential oil samples by agglomerative hierarchical cluster (AHC) analysis using the XLSTAT software, version 2015.4.01. Pearson correlation was selected as a measure of similarity, and the unweighted pair-group method with arithmetic average (UPGMA) was used for cluster definition.

### 2.5. Antifungal Screening

Antifungal activity was carried out using *Candida albicans* (ATCC #18804), *Cryptococcus neoformans* 24067 (serotype D or var. *neoformans*), and *Aspergillus niger* (ATCC #16888). Qualitative assessment of antifungal activity utilized broth macrodilution (for *C. albicans* and *C. neoformans*), whereas minimum inhibitory concentrations (MIC) were determined using microdilution methods. Initially cultures were grown on potato dextrose agar for 48–72 h before a single colony was isolated and grown in potato dextrose broth for 48–72 hours to create initial liquid cultures. Cells were diluted to a final concentration of 2 × 10^3^ cells/mL using MOPS (3-(*N*-morpholino)propanesulfonic acid) buffered RPMI (Roswell Park Memorial Institute) medium and 900 μL were aliquoted into sterile 12 × 75 mm tubes. Each *T. vulgaris* essential oil (100 μL of 1% DMSO solution) was added to each tube, which were then incubated at 37 °C for 72 h in a shaking incubator (175 rpm). For determination of the MIC for *C. albicans* and *C. neoformans*, microdilution in 96-well plates was performed in triplicate. Briefly, serial dilution of the *T. vulgaris* samples was performed by adding 50 µL of RPMI to each well then an equal volume of sample to be tested to the first row. After mixing, 50 µL was removed and added to the next row. The procedure was repeated for each row. To this mixture, 50 µL of cells diluted to 2000 cells/mL in RPMI were added to each well. The plates were incubated for 48 hours at 37 °C before growth was quantitated visually based on turbidity.

For the mold-like Ascomycota *A. niger*, disk diffusion was used to characterize each *T. vulgaris* essential oil. Initial cultures were grown on malt extract agar for 5–7 days before conidia were collected and suspended in potato dextrose broth. The suspension was then filtered into a sterile test tube using cheesecloth to remove hyphae. Conidia suspension was then diluted until it reached an OD_625_ of 0.1–0.2. The suspension (100 μL) was plated on malt extract agar before a sterile filter paper disk was placed in the center and 50 μL of *T. vulgaris* essential oil was added. The culture was grown for 4–5 days at 25 °C before zones of inhibition were determined.

## 3. Results and Discussion

### 3.1. Essential Oil Compositions

The chemical compositions of *T. vulgaris* essential oils from this study are listed in Table 1. *T. vulgaris* sample #1, a linalool chemotype collected from Nyons, France, was dominated by linalool (76.15%) and linalyl acetate (14.26%). The enantiomeric ratios of these two compounds were 1:99 d/l. Other compounds in the oil were β-caryophyllene (2.27%), camphor (1.79%, 100% d enantiomer), and camphene (1.17%, d/l ratio = 99:1). *T. vulgaris* #3 was a geraniol chemotype collected from Jablanicki, Serbia, and was rich in geraniol (59.75%) and esters of geraniol, geranyl acetate (16.72%) and geranyl propionate (1.26%). Oil #3 also had sizable concentrations of linalool (7.15%, d/l ratio = 97:3) and β-caryophyllene (3.67%). *T. vulgaris* #4 was collected from Pomoravje District, Serbia, and had sabinene hydrates as defining compounds with *cis*-sabinene hydrate (30.77%, d/l ratio = 97:3) and *trans*-sabinene hydrate (4.98%). Other major components in oil #4 were terpinene-4-ol (9.50%, d/l = 30:70), linalool (7.89%, d/l = 3:97), γ-terpinene (4.58%), and myrcene (4.09%). *T. vulgaris* #5, representing the thymol chemotype, was collected from Richerenches, France. The composition of oil #5 was rich in thymol (47.06%) and *p*-cymene (20.07%) with lesser quantities of linalool (5.00%, d/l = 1:99) and carvacrol (3.24%). Interestingly, the l-enantiomer dominated linalool in samples #1, #4, and #5, but was reversed in sample #3. Özek and co-workers have found l-linalool to be the predominant enantiomer in several *Thymus* species, but apparently they did not examine *T. vulgaris* [17]. Similarly, d-terpinen-4-ol was the major enantiomer in sample #3, but a minor one in samples #4 and #5. An enantiomeric composition analysis of *Melaleuca alternifolia* revealed this essential oil to be composed of mostly d-*cis*-sabinene hydrate and d-terpinen-4-ol [18]. d-α-Thujene was the dominant enantiomer in samples #3 and #4, while l-α-thujene predominated in sample #5. The l-enantiomer of α-thujene was found to be predominant in various *Citrus* essential oils [19]. l-α-Pinene dominated samples #1 and #3, but d-α-pinene was the major enantiomer in #4. Sample #5 showed a nearly racemic mixture of α-pinenes. l-Camphene dominated samples #1 and #3, but d-camphene dominated #4 and #5. Both d-α-pinene and d-camphene were found to be the predominant enantiomers in *Ocimum canum* and *O. kilimandscharicum* essential oils [20]. Remarkably, both d-camphor and d-borneol were the only enantiomers in sample #1, and d-borneol was also the only enantiomer found in samples #3 and #4. However, only l-camphor was found in essential oils #3 and #4. This is in contrast to the results of Tabanca and co-workers, who had exclusively observed the l-enantiomers of borneol along with 99% l-camphor in *Micromeria cristata* subsp. *phrygia* [21]. Thus, not only are the chemical profiles different between the different chemotypes, but the enantiomeric distributions are also profoundly different.

### 3.2. Chemotypes of Thymus vulgaris

**Figure 1:**
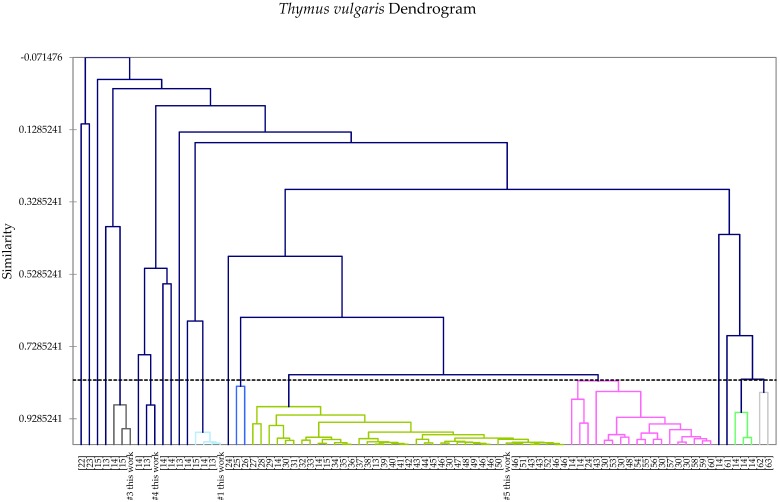
Dendrogram obtained from the agglomerative hierarchical cluster analysis of 85 *Thymus vulgaris* essential oil compositions.

A total of 85 *T. vulgaris* essential oil compositions were used to carry out a hierarchical cluster analysis (Figure 1). The cluster analysis revealed as many as 20 different chemotypes. The chemotype with the most samples (39) was the thymol chemotype, which had been recognized previously [12,13,14,15]. Other previously recognized chemotypes were the geraniol chemotype, with three representative samples (including sample #3 from Serbia in this study); the linalool chemotype, with four samples (including sample #1 from France in this study); the carvacrol chemotype, with two samples; the borneol chemotype, only one sample; the sabinene hydrate/terpinene-4-ol chemotype, with two samples (including sample #4 from Serbia in this study); and the cyclocitral/verbenol chemotype, with only one sample. The second most populated chemotype in the cluster analysis was a *p*-cymene/thymol type represented by 18 samples. The α-terpineol chemotype [12] was not found in the present study; only three *T. vulgaris* samples (all from Austria) showed α-terpineol concentrations around 10%, but all three had higher concentrations of other components [14]. Other chemotypes identified include carvacrol/γ-terpinene/thymol, which was previously described as carvacrol and thymol/carvacrol [14]; linalool/carvacrol/*p*-cymene, previously labeled carvacrol/linalool [14]; sabinene hydrate/geraniol/geranyl acetate/α-terpineol, previously described as geraniol/α-terpineol/sabinene hydrate [14]; sabinene hydrate/α-terpinyl acetate/thymol, previously described as α-terpineol/sabinene hydrate/thymol [14]; sabinene hydrate/linalool [14]; α-terpinyl acetate/carvacrol, previously described as α-terpineol/carvacrol [14]; carvacrol/α-terpineol/borneol; sabinene hydrate/ terpinen-4-ol, which includes one of the samples from France in this study; geranyl formate/geraniol, previously called geraniol [13]; *p*-cymene/thymol/carvacrol; terpinene-4-ol/*p*-cymene; camphor/camphene; and 1,8-cineole/α-terpinyl acetate.

### 3.3. Antifungal Activity

Three of the four *T. vulgaris* chemotypes in this study were tested for inhibition of *Aspergillus niger*, *Cryptococcus neoformans* var. *neoformans*, and *Candida albicans*. Macrodilution was utilized for the yeast-like Ascomycota, *C. albicans*, and Basidiomycota *C. neoformans*, to determine antifungal minimum inhibitory concentrations (MICs, Table 2). The *T. vulgaris* linalool and geraniol chemotypes both demonstrated some degree of inhibition against these pathogens. For the sporulating mold-like Ascomycota, *A. niger*, a larger surface area was required for hyphal growth. Thus, it was grown on malt extract agar plates with a filter disk impregnated with the *T. vulgaris* chemotype of interest. Disk diffusion showed only slight inhibition of *A. niger* with the only clear zone of inhibition for *T. vulgaris* sample #1. The remaining chemotypes showed growth over the filter disk, indicating no significant antifungal activity against *A. niger*. Because it has been shown that linalool is biotransformed to non-pathogenic compounds and that linalyl acetate increases *A. niger* hyphal growth [64], it is speculated that camphor in sample #1 is responsible for *A. niger* inhibition. Significant levels of camphor are not found in the other *T. vulgaris* samples.

The differential antifungal activities observed in this study mirror those previously reported by Giordani and co-workers [13]. That is, the sabinene hydrate chemotype showed the lowest antifungal activity, the linalool chemotype was next, then the geraniol chemotype. Giordani and co-workers had found that the thymol chemotype showed much stronger antifungal activity [13]. The *T. vulgaris* thymol chemotype was also found to be the most larvicidal against *Culex quinquefasciatus* [15] and exhibited the most antioxidant properties [14].

## 5. Conclusions

This work has presented the most comprehensive analysis of *Thymus vulgaris* chemotypes, revealing at least 20 different types based on essential oil composition. In addition, this is the first analysis to characterize the enantiomeric distributions of *T. vulgaris* monoterpenoids. Enantiomers are well known to elicit different odorant responses in insects [65,66] as well as humans [67], and it is reasonable to assume that different enantiomers will have different medicinal biological activities [68]. Thus, for example l-linalool has shown anticonvulsant activity in a mouse model whereas d-linalool was inactive [69]. Similarly, both d-α-pinene and d-β-pinene showed antifungal activity whereas the l-enantiomers were inactive [70], while l-α-pinene was more active than the d-enantiomer against *Listeria monocytogenes* [71]. Therefore, not only is the particular chemotype of a culinary and medicinal herb such as *T. vulgaris* an important consideration, but the enantiomeric distribution may also have a profound influence on its bioactivity, flavor, and aroma profile.

## Figures and Tables

**Table 1:** Chemical compositions and enantiomeric distributions of *Thymus vulgaris* essential oils.

RI	Compound	#1		#3		#4		#5	
		%	d/l	%	d/l	%	d/l	%	d/l
752	3-Methyl-1-penten-3-ol	tr							
783	Methyl α-methyl butyrate	0.10		0.07		tr		tr	
850	(3*Z*)-Hexenol							0.05	
922	Tricyclene	0.05		tr				0.09	
924	α-Thujene	tr		tr	91:9	0.71	87:13	0.43	10:90
932	α-Pinene	0.47	1:99	0.21	1:99	1.75	85:15	1.32	52:48
947	α-Fenchene							tr	
948	Camphene	1.17	99:1	0.38	99:1	0.25	0:100	1.19	10:90
971	Sabinene	tr		0.05	98:2	2.03	78:22		
977	1-Octen-3-ol	0.43		0.42					
977	1-Octen-3-ol + β-Pinene					0.97	20:80	0.54	20:80
983	6-Methylhept-5-en-2-one			0.05					
983	3-Octanone					tr		tr	
988	Myrcene	0.09		0.44		4.09		1.59	
996	3-Octanol	tr		0.07		tr			
1004	(3*Z*)-Hexenyl acetate	tr							
1004	*p*-Mentha-1(7),8-diene							tr	
1006	α-Phellandrene					tr	55:45	0.11	
1008	δ-3-Carene							tr	
1016	α-Terpinene			tr		2.65		1.30	
1019	*o*-Cymene							0.06	
1024	*p*-Cymene	0.09		0.18		1.09		**20.07**	
1028	Limonene	0.05		0.39	85:15	2.85	86:14	0.39	80:20
1030	β-Phellandrene			tr		0.37		0.09	
1031	1,8-Cineole	0.37		0.31		0.30		0.72	
1033	*m*-Cymene							tr	
1034	Lavender lactone + (*Z*)-β-Ocimene	tr							
1045	(*E*)-β-Ocimene	tr						0.05	
1057	γ-Terpinene			0.09		4.58		9.03	
1063	3-Methylbut-2-enyl butanoate	tr							
1069	*cis*-Sabinene hydrate			0.31		**30.77**	**97:3**	0.17	
1070	*cis*-Linalool oxide (furanoid)	0.28							
1071	Pinol							tr	
1084	Terpinolene					0.97		0.07	
1085	*trans*-Linalool oxide (furanoid)	0.23						tr	
1089	*p*-Cymenene							tr	
1099	Linalool	**76.15**	1:99	7.15	97:3	7.89	3:97	5.00	1:99
1101	*trans*-Sabinene hydrate			tr		4.98		tr	
1103	Hotrienol	0.07							
1104	Nonanal	0.05				tr			
1106	α-Pinene oxide			tr					
1107	1-Octen-3-yl acetate	tr				tr			
1124	*cis-p-*Menth-2-en-1-ol					0.65			
1136	*trans*-Limonene oxide					tr			
1142	*trans-p*-Menth-2-en-1ol					0.25			
1147	Camphor	1.79	100:0	0.11	0:100	0.10	0:100	1.42	95:5
1148	α-Cyclogeraniol			tr					
1154	β-Pinene oxide					0.05			
1162	Lavandulol	tr		tr				tr	
1169	*cis*-Linalool oxide (pyranoid)	tr							
1171	Borneol	0.40	100:0	1.00	100:0	0.28	100:0	1.50	71:29
1174	*trans*-Linalool oxide (pyranoid)	tr							
1178	p-Mentha-1,8-dien-4-ol					tr			
1180	Terpinen-4-ol	0.06		0.17	70:30	9.50	30:70	1.25	40:60
1184	(3*Z*)-Hexenyl butanoate	tr							
1186	*p*-Cymen-8-ol							tr	
1194	α-Terpineol	0.11	65:35	0.09	65:35	2.69	91:9	0.16	60:40
1196	*cis*-Piperitol					0.14			
1197	*cis*-Dihydrocarvone	tr		tr				tr	
1205	β-Cyclogeraniol			tr					
1206	Decanal	tr							
1208	*trans*-Piperitol					0.16			
1217	7-Ethylidenebicyclo[3.3.0]octan-3-one					0.90			
1220	6,7-Epoxyneral			tr					
1223	Nerol	tr		0.97					
1223	7-Methylenebicyclo[3.3.1]nonan-3-ol					6.07			
1225	Citronellol	tr		0.35					
1228	Thymol methyl ether							0.19	
1230	6,7-Epoxygeranial			tr					
1234	4-*t*-Amylcyclohexanone	0.08							
1237	Neral	tr		0.61		tr			
1237	Carvacrol methyl ether							0.32	
1243	Carvone			tr				0.09	
1249	Linalyl acetate	14.26	1:99			3.40	0:100		
1249	Geraniol			**59.75**		0.24		0.05	
1266	Geranial	tr		1.25		tr		tr	
1271	(2*E*)-Decenal	tr							
1279	Isothymol							0.07	
1282	Lavandulyl acetate	tr							
1284	Bornyl acetate	0.21		0.14		0.07			
1284	*neo-iso*-3-Thujanol acetate							tr	
1288	Thymol			0.42		tr		**47.06**	
1296	Carvacrol	0.05						3.24	
1296	Geranyl formate			0.09					
1331	2,3-Epoxygeraniol			0.06					
1341	*cis-p*-Menthadienyl acetate			0.19		4.75			
1344	Citronellyl acetate					0.28			
1346	α-Terpinyl acetate					0.05			
1348	Citronellyl acetate			0.07					
1349	Eugenol							0.08	
1357	Neryl acetate	tr		0.18		tr			
1372	*trans-p*-Menthadienyl acetate					0.05			
1375	α-Copaene							tr	
1377	Geranyl acetate	0.06		16.72		0.48			
1383	α-Bourbonene	0.20		tr		0.09			
1388	β-Elemene	tr							
1403	Isocaryophyllene	tr		tr					
1419	β-Caryophyllene	2.27		3.67		2.03		1.79	
1429	β-Copaene	tr							
1429	*cis*-Carvyl propanoate					0.30			
1434	7-Methyl-3-methylene-7-octen-1-yl propanoate					tr			
1437	Aromadendrene							tr	
1454	α-Humulene	0.06		0.12		0.06		0.05	
1468	Geranyl propanoate	tr		1.26					
1473	*trans*-Cadina-1(6),4-diene							0.06	
1480	Germacrene D	0.26		0.05		0.58			
1489	Viridiflorene							tr	
1494	Bicyclogermacrene			tr		0.09			
1497	α-Muurolene							tr	
1506	Geranyl isobutyrate			0.15					
1506	β-Bisabolene							tr	
1511	δ-Amorphene							0.07	
1516	δ-Cadinene	tr						0.16	
1531	*trans*-Cadina-1,4-diene							tr	
1547	Elemol			0.96					
1554	Geranyl butanoate	0.05		0.74					
1575	Germacrene-d-4-ol	0.10				tr			
1575	Spathulenol			tr					
1581	Caryophyllene oxide	0.41		0.50		0.10		0.13	
1595	Geranyl isovalerate			0.06					
1631	10-*epi*-γ-Eudesmol			tr					
1654	α-Eudesmol			0.17					

**Table 2:** Antifungal activities (minimum inhibitory concentrations, MIC) of *Thymus vulgaris* essential oils.

Essential Oil	MIC (μg/mL)	
	*C. albicans*	*C. neoformans*
#1 (linalool chemotype)	1250	313
#3 (geraniol chemotype)	625	156
#4 (sabinene hydrate chemotype)	>2500	>2500
